# HKDC1 promotes the tumorigenesis and glycolysis in lung adenocarcinoma via regulating AMPK/mTOR signaling pathway

**DOI:** 10.1186/s12935-020-01539-7

**Published:** 2020-09-12

**Authors:** Xinyu Wang, Bowen Shi, Yue Zhao, Qijue Lu, Xiang Fei, Chaojing Lu, Chunguang Li, Hezhong Chen

**Affiliations:** Department of Thoracic Surgery, Changhai Hospital, Second Military Medical University, Shanghai, 200433 China

**Keywords:** HKDC1, Tumorigenesis, Lung adenocarcinoma, AMPK/mTOR signaling pathway

## Abstract

**Background:**

Hexokinase domain component 1 (HKDC1) plays an oncogenic role in certain types of cancer, such as lymphoma, liver cancer, and breast cancer. Previous bioinformatics study revealed that HKDC1 was significantly upregulated in lung adenocarcinoma (LUAD). However, its biological functions and potential mechanism in LUAD have not been studied.

**Methods:**

We performed bioinformatics analysis, quantitative real-time polymerase chain reaction (qRT-PCR), western blotting, immunohistochemistry, and a series of functional assays in vitro and in vivo to investigate the roles of HKDC1 in LUAD.

**Results:**

We discovered that HKDC1 was highly expressed in LUAD tissues and cell lines, and the positive expression of HKDC1 was correlated with aberrant clinicopathological characteristics in LUAD patients. Furthermore, HKDC1 could serve as a prognostic predictor for LUAD patients. Overexpression of HKDC1 promoted proliferation, migration, invasion, glycolysis, EMT and tumorigenicity, whereas knockdown of HKDC1 produced the opposite functional effects. Mechanistically, HKDC1 could regulate the AMPK/mTOR signaling pathway to perform its biological function.

**Conclusions:**

Our findings suggest that HKDC1 plays an oncogenic role in LUAD. Targeting this gene may provide a promising therapeutic target to delay LUAD progression.

## Background

Lung cancer ranks first among all malignant tumors in terms of morbidity and mortality, posing a major threat to human health [1, 2]. Lung adenocarcinoma (LUAD) is one of the most common subtypes of lung cancer, accounting for 30–35% of primary lung cancers [3]. Although tremendous efforts have been devoted to fighting against lung cancer, limited improvement in survival has been achieved. Recently, molecular targeted therapy has shown promising results in treating LUAD [4–6], prompting researchers to explore new molecular mechanisms in LUAD.

Aerobic glycolysis, also known as Warburg effect, refers to the conversion of glucose into lactate in cancer cells even under sufficient oxygen conditions [7]. Accumulating evidence has demonstrated that glycolysis contributes to tumor growth and metastasis through such unique metabolic pathway. Herein, it is a new strategy to delay tumor progression by inhibiting glycolysis in cancer cells [8].

Hexokinases (HKs) are a family of enzymes that catalyze the first step in glucose metabolism by phosphorylating glucose to glucose-6-phosphatase of glucose utilization [9]. So far, four HK isozymes have been identified in mammals, named from HK I to HK IV. Among the four isoforms of HKs, HK2 is the most widely studied and a key player in aerobic glycolysis in cancers. Overexpression of HK2 is also observed in liver cancer [10], colorectal cancer [11], prostate cancer [12], and esophageal squamous cell carcinoma [13], and is associated with poor prognosis in patients. Moreover, HK2 is also reported to be upregulated and to function as a novel oncogene in lung cancer [14, 15].

Hexokinase domain component 1 (HKDC1) is a recently discovered protein, that is categorized as a putative fifth hexokinase [16]. Under physiological conditions, HKDC1 is extremely critical to maintain whole-body glucose homeostasis [17–19]. However, aberrant expression of HKDC1 contributes to the progression of certain types of diseases and cancers. For example, overexpression of HKDC1 could lead to the metabolic dysfunction of hepatocytes, which may be associated with nonalcoholic fatty liver disease [20]. In addition, increasing evidence shows that HKDC1 may play oncogenic roles in cancers, such as lymphoma [21], liver cancer [22], breast cancer [23] and colorectal cancer [24], indicating that HKDC1 may serve as a therapeutic target in cancers.

Previous bioinformatics analysis predicted that HKDC1 could be a promising therapeutic target for lung cancer [25]. However, this hypothesis remains to be validated, and the downstream mechanism of HKDC1 in lung cancer needs to be explored. In this study, we performed a series of functional assays in vitro and in vivo to investigate the roles of HKDC1 in LUAD.

## Methods

### Cell culture

The LUAD cell lines A549 and H1299, and the normal human bronchial epithelial cell line HBE were obtained from the Chinese Academy of Sciences Cell Bank (Shanghai, China) and cultured in Dulbecco’s modified Eagle medium (DMEM) (Invitrogen, Carlsbad, CA) containing 10% fetal bovine serum (FBS) (Invitrogen) at 37 °C in a humidified atmosphere of 5% CO_2_.

### Clinical tissue and specimens

A total of 20 fresh primary LUAD tissues and paired adjacent normal tissues were obtained from surgeries at Changhai Hospital, Second Military Medical University (Shanghai, China). Seventy-five paired paraffin-embedded LUAD specimens used in this study were collected from patients in 2013 who were diagnosed with primary LUAD and none of them received preoperative chemotherapy or radiotherapy. Overall survival (OS) was defined as the interval between surgery and death or last observation. This study was approved by the Ethics Committee of Changhai Hospital. All patients provided written informed consent upon enrolment.

### Construction of reagents for gene overexpression and knockdown

We constructed lentiviral vectors encoding the human HKDC1 gene or green fluorescent protein (GFP) in the pLenti-EF1a-EGFP-P2A-Puro-CMV-MCS-3Flag vector (HeYuan Bio-technology Co., Shanghai, China), which were named ov-HKDC1 or ov-NC. Stable LUAD cell knockdown of HKDC1 was generated using lentiviral constructs expressing sh-HKDC1 (sh-HKDC1#1: GGTGGACAGGTTCCTGTAT), sh-HKDC1#2: GGTCAGTGCGAATGTACAA) or sh-NC. LUAD cells were infected for 48 h and then selected with puromycin. Stable LUAD cell lines were successfully established if the infection efficiency was > 85%.

### Immunohistochemistry (IHC)

The LUAD tissue slides were incubated with anti-HKDC1 (1:200, Abcam, ab228729) primary antibody. IHC scoring was performed using a modified Histo-score (H-score) by two independent pathologists. Briefly, the proportion of positively stained cells was scored as 0–100% (< 25% scored 0, 25–50% scored 1, 50–75% scored 2, 75–100% scored 3) and the intensity score was scored as 0 (negative), 1+ (weakly positive), 2+ (moderately positive), or 3+ (strongly positive). A final score was then calculated by multiplying these two scores.

### RNA extraction and quantitative RT-PCR (qRT-PCR)

Total RNA was extracted from cultured LUAD cell lines or tissue specimens using TRIzol (Invitrogen, Grand Island, NY) according to the manufacturer’s instructions. The cDNA was synthesized using the PrimeScript RT Reagent Kit (TaKaRa Bio, Shiga, Japan) following the manufacturer’s instructions. Real-time PCR was performed on a Roche Light Cycler 480 (Roche) using SYBR Green PCR Master Mix (TaKaRa Bio, Shiga, Japan). The fold change relative to the mean value was determined by 2^−ΔΔCt^. All experiments were performed in triplicate. Primer sequences are listed as follows.

HKDC1:

5′-ATCCTGGCAAGCAGAGATACG-3′ (forward).

5′-GACGCTCTGAAATCTGCCCT-3′ (reverse);

GAPDH:

5′-GGAGCGAGATCCCTCCAAAAT-3′ (forward).

5′-GGCTGTTGTCATACTTCTCATGG-3′ (reverse);

### Western blotting

Whole cultured cells were homogenized in 0.1% SDS and 1 mM PMSF. Protein extracts were subjected to SDS-PAGE and analyzed using the following primary antibodies against the following antigens: HKDC1 (Abcam, ab228729), phospho-AMPKα (Cell Signaling Technology, 2535), phospho-mTOR (Cell Signaling Technology, 5536), phospho-p70S6 (Cell Signaling Technology, 9234), Vimentin (Santa Cruz, sc-6260), Snail (CST, 3879), E-cadherin (Abcam, ab40772), N-cadherin (Abcam, ab18203) and GAPDH (Abcam, ab8245). Then, the membranes were incubated with secondary antibodies (CST, 7076, 7074) at room temperature for 1 h. All experiments were performed in triplicate. The results of western blotting bands were quantified by measuring the gray values (ImageJ software, Rawak Software Inc, Stuttgart, Germany).

### Reagents

AICAR (CST, 9944) was used to activate AMPK signaling and rapamycin (Abcam, ab120224) was used to inhibit mTOR signaling in LUAD cells. LUAD cells were incubated at 37 °C for 24 h with 3 mM AICAR and 100 μM rapamycin for subsequent experiments.

### Bioinformatics analysis

We utilized the Oncomine (https://www.oncomine.org/resource/login.html) and UALCAN (https://ualcan.path.uab.edu/analysis.html) databases to detect the expression level of HKDC1 genes.

### Measurement of glucose and lactate

Transfected A549 and H1299 cells were seeded in 6-well plates (5 × 10^5^) and the culture media were harvested 48 h after transfection. The glucose and lactate levels were measured using a Glucose Assay Kit (Sigma-Aldrich, USA) and a Lactic Acid Assay Kit (Sigma-Aldrich, USA), respectively, according to the manufacturer’s protocol. The values were normalized to the total protein concentration. All experiments were performed in triplicate.

### Cell proliferation assays

Cell viability was measured by Cell Counting Kit-8 (CCK-8, Bimake, USA). Briefly, transfected cells were seeded in 96-well plates (5000 cells/well) and cultured for 3 days to assess proliferation. The absorbance was measured at 450 nm. All experiments were performed in triplicate.

### Cell migration and invasion assay

Cell migration and invasion ability was assessed by 24-well Transwell chambers (Corning) with or without a Matrigel (Corning, Bedford, MA, USA) coating. Briefly, approximately 1 * 10^5^ cells were resuspended in 300 µL serum-free DMEM and seeded into the upper chambers, whereas the bottom chamber was filled with 500 µL 10% FBS medium. Twenty-four hours later, the migrated/invaded cells in the lower chamber were fixed with 4% paraformaldehyde and stained with 1% crystal violet. All experiments were performed in triplicate. Cell counting was accomplished by ImageJ software (Rawak Software Inc, Stuttgart, Germany).

### Xenograft mice

Five-week-old male BALB/c nude mice were purchased from Shanghai Experimental Center (Shanghai, China). Xenograft tumor models were established by subcutaneous injection of ovHKDC1 or shHKDC1 (5 × 10^6^) transfected A549 cells into the right dorsal flank. Three mice were randomly assigned to each group. One week after injection, the mice were measured once a week for a total of 4–5 weeks. Tumor volume (V, cm^3^) was evaluated by a slide caliper (Shanghai, China) based on tumor length (L) and width (W) with the following formula: V = 1/2 × L × W^2^. All manipulations were performed in accordance with currently prescribed guidelines and under a protocol approved by the SMMU Ethical Review Committee (Shanghai, China).

### Statistical analysis

Data analysis was carried out using IBM SSPS 24 (IBM Corp., Armonk, NY, USA) and data were reported as the mean ± standard deviation (mean ± SD). Student’s t-test was used to determine differences between groups and two-tailed ANOVA was performed in cases of multiple groups. The association between genes and clinicopathological features was analyzed by the Chi-square test or Fisher’s exact test. Kaplan–Meier curves were used to compare the OS between groups. Multivariate analysis was performed to determine independent factors affecting the prognosis of patients. Differences were considered statistically significant when p < 0.05.

## Results

### HKDC1 was upregulated in LUAD tissues and cells

**Fig.1:**
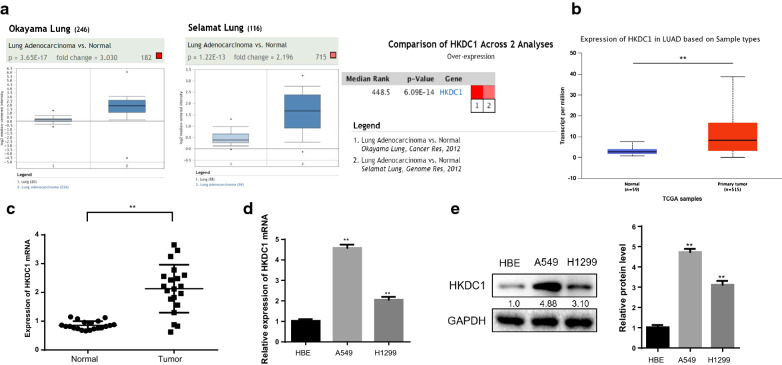
HKDC1 was upregulated in LUAD tissues and cells. **a**, **b** Oncomine (**a**) and UALCAN (**b**) data showed that HKDC1 was highly expressed in LUAD tissues compared with normal tissues. **c** The HKDC1 mRNA expression level was significantly elevated in LUAD tissues compared with normal lung tissues in 20 paired fresh tissues. **d**, **e** The HKDC1 expression level was obviously higher in the LUAD cell lines than in the normal human lung epithelial cell line HBE on mRNA (**d**) and protein level (**e**). Data were expressed as the mean ± standard deviation (mean ± SD) **p < 0.01

First, we measured the expression of HKDC1 in two public databases, Oncomine and UALCAN. The data of Okayama lung and Selamat lung samples from the Oncomine database and TCGA samples from the UALCAN database showed that the expression level of HKDC1 was significantly elevated in LUAD tissues compared with normal lung tissues (Fig. 1a, b).

To further confirm the expression of HKDC1 in LUAD, we examined the expression of HKDC1 mRNA in 20 fresh LUAD tissues and their corresponding adjacent normal tissues. The HKDC1 mRNA expression level was significantly elevated in LUAD tissues compared to normal lung tissues (p < 0.01, Fig. 1c). Simultaneously, we examined HKDC1 expression in cell lines by qRT-PCR analysis and western blotting. The HKDC1 expression level was obviously upregulated in the LUAD cell lines compared with the normal human lung epithelial cell line HBE (Fig. 1d, e).

### HKDC1 was associated with aggressive features and poor prognosis in LUAD

**Fig. 2:**
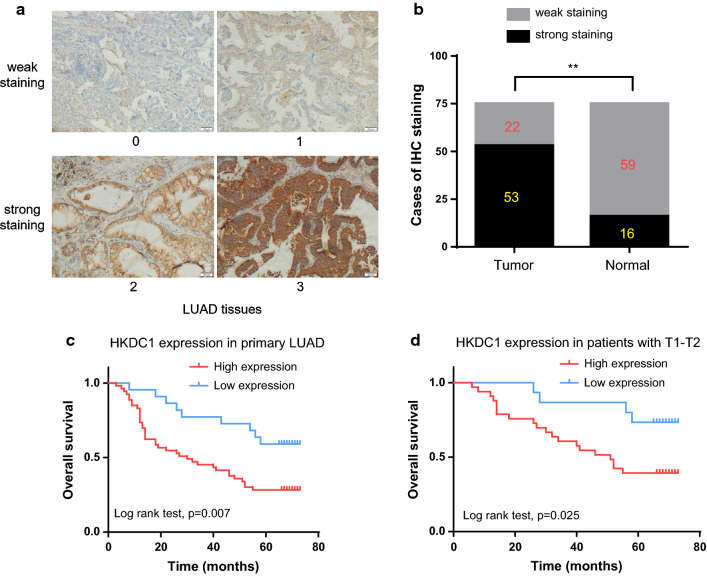
HKDC1 was associated with aggressive features and poor prognosis in LUAD. **a** Representative staining intensity of HKDC1 was graded from 0 to 3 by IHC analysis (magnification: ×200). **b** The ratio for strong staining and weak staining in 75 paired tumor and non-tumor tissues. **c** Overall survival was poorer in patients with high HKDC1 than in those with low expression among 75 LUAD patients. **d** Subgroup analysis demonstrated that pT1–T2 stage patients with high expression of HKDC1 had unfavorable overall survival. **p < 0.01

To explore the expression pattern of HKDC1 in LUAD, we performed IHC in 75 paired LUAD specimens. As shown in Fig. 2a, the expression level of HKDC1 in LUAD was graded from 0 to 3 by the IHC staining intensity. Strong staining of HKDC1 was far more frequently observed in LUAD tissues (53/75, 70.7%) than in adjacent normal tissues (16/75, 21.3%, Fig. 2b). Table 1 showed the correlation between HKDC1 expression and clinicopathological features of LUAD patients. The results demonstrated that the expression of HKDC1 was associated with histologic differentiation (p = 0.003), and pN stage (p = 0.009) while there was no association between HKDC1 expression and age (p = 0.681), sex (p = 0.302), tumor location (p = 0.428), pT stage (p = 0.627) or TNM stage (p = 0.843).

**Table 1:** Correlations between HKDC1 expression and clinicopathologic features

Characteristic	HKDC1 expression		p value
	Low expression (%)	High expression (%)	
Age			0.681
> 55	16 (30.77%)	36 (69.23%)	
≤ 55	6 (26.09%)	17 (73.91%)	
Sex			0.302
Male	10 (24.39%)	31 (75.61%)	
Female	12 (35.29%)	22 (64.71%)	
Tumor location			0.428
Left lung	9 (25.00%)	27 (75.00%)	
Right lung	13 (33.33%)	26 (66.67%)	
Histologic differentiation			*0.003*
Well + moderate	20 (40.82%)	29 (59.18%)	
Poor	2 (7.69%)	24 (92.31%)	
Pathological T stage			0.627
T1–2	15 (31.25%)	33 (68.75%)	
T3–4	7 (25.93%)	20 (74.07%)	
Pathological N stage			*0.009*
N0	16 (43.24%)	21 (56.76%)	
N+	6 (15.79%)	32 (84.21%)	
TNM stage			0.843
I + II	13 (30.23%)	30 (69.77%)	
III + IV	9 (28.13%)	23 (71.87%)	

By Kaplan–Meier analysis, overall survival (OS) was more unfavorable in patients with high expression of HKDC1 than in those with low expression (p = 0.007, Fig. 2c). Further subgroup analysis demonstrated that high expression of HKDC1 was associated with an unfavorable OS in pT1–T2 stage patients (Fig. 2d). By univariate Cox analysis, differentiation, pT stage, pN stage and HKDC1 expression showed statistically significant associations with OS (p = 0.001; p < 0.001; p < 0.001; p = 0.010, respectively). Multivariate Cox analysis revealed that HKDC1 expression was still an independent factor affecting OS (p = 0.018, Table 2).

**Table 2:** Univariate and multivariate survival analysis for patients with LUAD

Characteristic	HR	95% CI	p value
Univariate analysis			
Age (≤ 55 vs > 55)	1.277	0.697–2.338	0.428
Sex (male vs female)	1.677	0.930–3.024	0.086
Location (left vs right)	1.582	0.891–2.811	0.118
Differentiation (poor vs well + moderate)	2.682	1.501–4.794	*0.001*
pT stage (T3–4 vs T1–2)	3.259	1.820–5.836	< *0.001*
pN stage (N+ vs N0)	5.835	3.007–11.323	< *0.001*
HKDC1 expression (high vs low)	2.625	1.265–5.448	*0.010*
Multivariate analysis			
Differentiation (poor vs well + moderate)	1.276	0.644–2.528	0.485
pT stage (T3–4 vs T1–2)	2.245	1.185–4.253	*0.013*
pN stage (N+ vs N0)	4.561	2.062–10.089	< *0.001*
HKDC1 expression (high vs low)	2.598	1.176–5.739	*0.018*

*95% CI* = 95% confidence interval; *HR* = hazard risk

### HKDC1 promoted proliferation, invasion, and migration in vitro and LUAD cell tumorigenesis in vivo

**Fig. 3:**
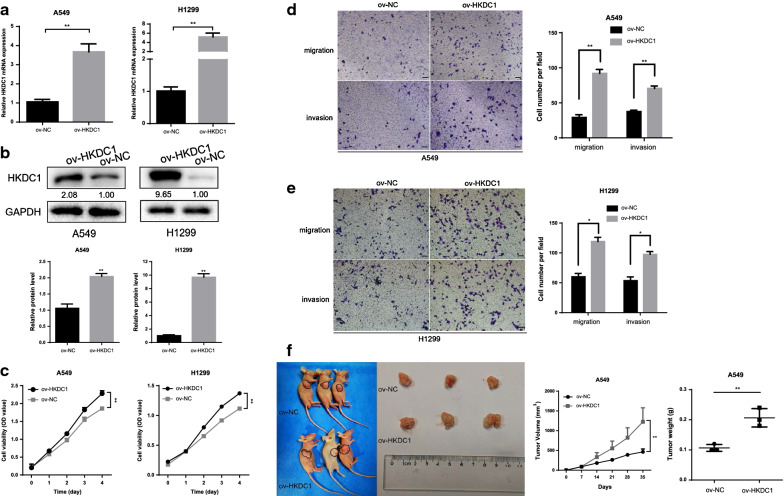
HKDC1 promoted proliferation, invasion, migration in vitro and tumorigenesis in vivo. **a**, **b** The mRNA (**a**) and protein levels (**b**) of HKDC1 were detected after transfection with overexpressed HKDC1 in both A549 and H1299 cells. **c** The viability of LUAD cells with HKDC1 overexpression was increased by CCK-8 assay. **d**, **e** Overexpression of HKDC1 promoted A549 (**d**) and H1299 (**e**) cells migration and invasion in vitro as assessed by the wound healing assay (scale bar, 100 μm). **f** HKDC1 overexpression promoted tumor growth in vivo. Data were expressed as the mean ± standard deviation (mean ± SD) *p < 0.05, **p < 0.01

To investigate the biological behaviors of HKDC1 in LUAD cells, we established two LUAD cell lines stably expressing HKDC1, and the expression level of HKDC1 was examined by qRT-PCR and western blotting. The results showed that HKDC1 expression was obviously upregulated in ov-HKDC1 cells compared to the control cells (Fig. 3a, b).

To determine the effect of HKDC1 on cell proliferation, we performed a CCK-8 assay. The results indicated that HKDC1 overexpression promoted the proliferative ability of LUAD cells (Fig. 3c). Subsequently, a Transwell assay was performed to investigate the impact of HKDC1 on cell migration and invasion. The results revealed that HKDC1 overexpression in A549 and H1299 cells increased the migratory and invasive abilities compared to those of the corresponding control cells (Fig. 3d, e). To investigate the effect of HKDC1 on LUAD tumor growth in vivo, we performed xenograft growth assays in nude mice. As a result, the tumors in the HKDC1 overexpression group were obviously larger and heavier than those in the control group (Fig. 3f).

### Silencing HKDC1 inhibited the proliferation, invasion, and migration of LUAD

**Fig. 4:**
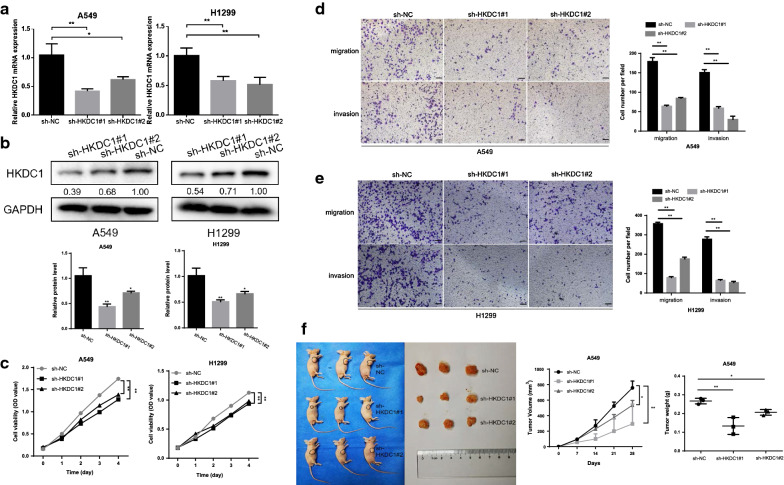
Silencing HKDC1 inhibited proliferation, invasion, and migration in vitro and tumorigenesis in vivo. **a**, **b** The mRNA (**a**) and protein level (**b**) of HKDC1 was detected after transfected with sh-HKDC1 in both A549 and H1299 cells. **c** The viability of LUAD cells with sh-HKDC1 was decreased by CCK-8 assay. **d**, **e** Knockdown of HKDC1 inhibited A549 (**d**) and H1299 (**e**) cells migration and invasion in vitro as assessed by the wound healing assay (scale bar, 100 μm). **f** HKDC1 knockdown inhibited tumor growth in vivo. Data were expressed as the mean ± standard deviation (mean ± SD) *p < 0.05, **p < 0.01

Next, we constructed the LUAD cells with lentivirus-mediated HKDC1 knockdown, which was validated by qRT-PCR and western blotting (Fig. 4a, b). The results revealed that knockdown of HKDC1 inhibited the proliferation of LUAD cells and decreased the migratory and invasive abilities compared to those in the corresponding control cells (Fig. 4c–e). In addition, animal experiments showed that the tumors of the HKDC1 knockdown groups were significantly smaller and lighter than those of the control group, as expected (Fig. 4f).

### HKDC1 regulated glycolysis and epithelial–mesenchymal transition in LUAD cells

**Fig. 5:**
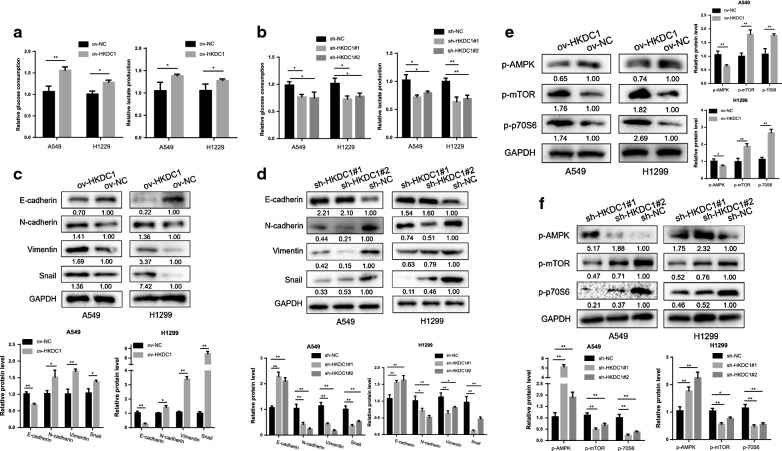
HKDC1 regulated glycolysis, epithelial–mesenchymal transition, and AMPK/mTOR signaling pathway in LUAD cells. **a** Overexpression of HKDC1 promoted glucose consumption and lactate production in both A549 and H1299 cells. **b** Silencing HKDC1 reduced glucose consumption and lactate production in both A549 and H1299 cells. **c** Overexpression of HKDC1 promoted the expression of N-cadherin, Snail, and Vimentin while decreased the expression of E-cadherin in LUAD cells. **d** Silencing HKDC1 decreasing the expression of N-cadherin, Snail, and Vimentin while increasing the expression of E-cadherin in LUAD cells. **e**, **f** HKDC1 regulated the expression of p-AMPK, p-mTOR and p-p70S6 in LUAD cells. Data were expressed as the mean ± standard deviation (mean ± SD) *p < 0.05, **p < 0.01

HK enzymes are rate-limiting enzymes involving in regulating glycolysis in multiple cancers [26]. As HKDC1 is a member of the HK family, it is necessary to investigate the effects of HKDC1 on glycolysis. Glucose consumption and lactate production are two common measures to reflect glycolysis levels [27]. First, we detected the glycolysis levels by overexpressing HKDC1 in LUAD cells. The results showed that both glucose consumption and lactate production were significantly increased in ov-HKDC1 A549 and H1299 cells compared with the control cells (Fig. 5a). In contrast, silencing HKDC1 reduced the glucose consumption and lactate production in both A549 and H1299 cells (Fig. 5b). Taken together, these data demonstrated that HKDC1 promoted aerobic glycolysis and lactate production in LUAD cells.

Increasing studies have shown that aberrant cancer metabolism can regulate EMT through specific pathological pathways [28], and in turn, EMT can also exacerbate the dysregulation of glucose metabolism. Therefore, we evaluated the impact of HKDC1 on EMT in LUAD cells. The results of western blotting revealed decreased levels of the epithelial marker E-cadherin and increased levels of mesenchymal markers such as N-cadherin, Vimentin, and Snail in the ov-HKDC1 group (Fig. 5c). In contrast, increased epithelial marker expression and decreased mesenchymal marker were observed in the sh-HKDC1 group (Fig. 5d), indicating that HKDC1 could enhance the EMT capacity of LUAD cells.

### HKDC1 regulates AMPK/mTOR signaling pathway

To gain further insight into the molecular mechanism by which HKDC1 mediated oncogenic roles in LUAD, we attempted to identify the potential signaling pathway of HKDC1. PI3K/AKT/mTOR and AMPK/mTOR pathways are two important signaling cascades that are involved in regulating aerobic glycolysis of cancer cells [29]. Thus, the impacts of HKDC1 on the PI3K/AKT/mTOR and AMPK/mTOR signal pathway were subsequently investigated. The results showed that the phosphorylated AMPKα at Thr172 was markedly decreased, and the phosphorylated mTOR at Ser2448 and phosphorylated p70S6 were significantly increased in ov-HKDC1 LUAD cells (Fig. 5e), whereas the p-AMPK level was increased and p-mTOR and p-p70S6 level were decreased in sh-HKDC1 LUAD cells (Fig. 5f), indicating that HKDC1 acted as an important regulator on the AMPK/mTOR signal pathway. However, the levels of PI3K, p-AKT and total AKT remained unchanged after HKDCI was altered (data not shown), which indicated that HKDC1 had no obvious effects on PI3K/AKT.

**Fig. 6:**
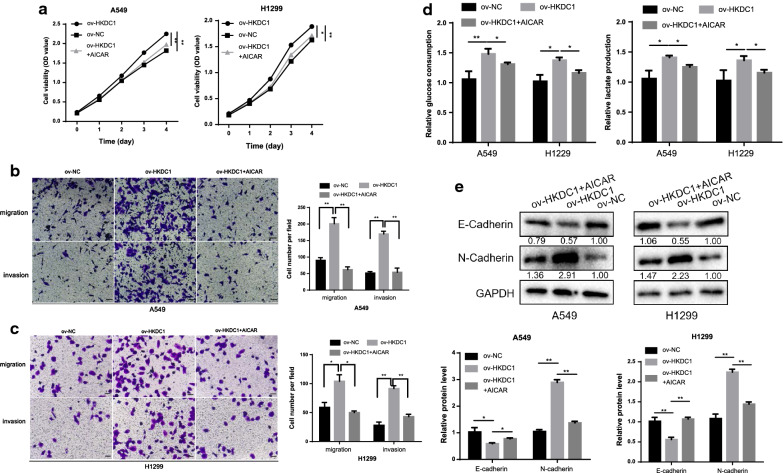
AICAR attenuated the oncogenic effects of HKDC1 in LUAD cells. **a** AICAR attenuated the proliferative ability of HKDC1 in LUAD cells. **b**, **c** AICAR attenuated the migratory and invasive ability of HKDC1 in LUAD cells (scale bar, 100 μm). **d** AICAR attenuated the glycolytic effect of HKDC1 in LUAD cells. **e** AICAR attenuated the EMT effect of HKDC1 in LUAD cells. Data were expressed as the mean ± standard deviation (mean ± SD) *p < 0.05, **p < 0.01

**Fig. 7:**
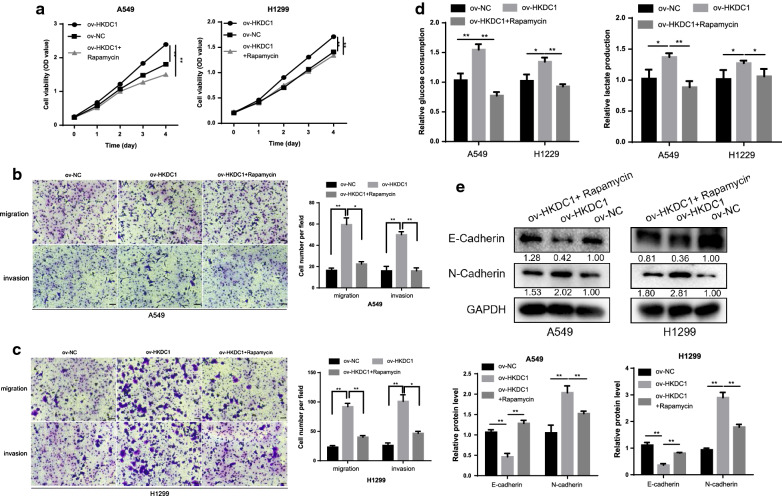
Rapamycin attenuated the oncogenic effects of HKDC1 in LUAD cells. **a** Rapamycin attenuated the proliferative ability of HKDC1 in LUAD cells. **b**, **c** Rapamycin attenuated the migratory and invasive ability of HKDC1 in LUAD cells (scale bar, 100 μm). **d** Rapamycin attenuated the glycolytic effect of HKDC1 in LUAD cells. **e** Rapamycin attenuated the EMT effect of HKDC1 in LUAD cells. Data were expressed as the mean ± standard deviation (mean ± SD) *p < 0.05, **p < 0.01

To further verify whether the oncogenic role of HKDC1 was mediated by the AMPK/mTOR signal pathway in LUAD, we performed rescue experiments. AICAR (an activator of AMPK signaling) and rapamycin (an inhibitor of mTORC1 signaling) were added to ov-HKDC1 LUAD cells. According to the results of a series of functional experiments, both AICAR and rapamycin attenuated the abilities of proliferation (Figs. 6a, 7a), migration, invasion (Figs. 6b, c, 7b, c), glycolysis (Figs. 6d, 7d) and EMT (Figs. 6e, 7e) in ov-HKDC1 LUAD cells.

Collectively, these results showed that AICAR and rapamycin significantly decreased HKDC1-induced oncogenic functions of LUAD cells, indicating that HKDC1 affected biological behaviors via regulating AMPK/mTOR pathway.

## Discussion

In this study, we discovered that HKDC1 was highly expressed in LUAD tissues and cell lines, and the positive expression of HKDC1 was correlated with aberrant clinicopathological characteristics in LUAD patients. In addition, HKDC1 could serve as an OS predictor for LUAD patients. Overexpression of HKDC1 promoted the proliferation, migration, invasion, glycolysis, EMT and tumorgenicity in vitro and in vivo, whereas knockdown of HKDC1 produced the opposite functional effects. Mechanistically, HKDC1 could regulate AMPK/mTOR signaling pathway to perform its biological function. Targeting HKDC1 may provide a new therapeutic strategy for LUAD treatment.

Li and colleagues screened the top 20 genes with potential of being developed into candidate therapeutic targets of lung cancer, among which 19 genes were targeted by approved drugs or drugs used in clinical trials [25]. HKDC1 was the only one that was not included in these 20 candidate targets, which attracted our interest. Moreover, the role of HKDC1 as an oncogene in liver cancer and colorectal cancer prompted us to investigate its functional effect in LUAD. In the present study, we found that HKDC1 was highly expressed in LUAD tissues while was not expressed or lowly expressed in adjacent normal lung epithelial tissues, and its expression was correlated with aberrant clinicopathological characteristics and prognosis. These data preliminarily revealed that HKDC1 had an oncogenic function in LUAD and these unique properties make it a perfect therapeutic target for LUAD.

Metabolic reprogramming, such as glycolysis under aerobic conditions, has been increasingly deemed a hallmark for tumor progression in multiple cancers [30]. Elevated glycolysis in cancer cells promotes glucose uptake and lactate production to fulfil their metabolic demands, followed by the increased invasion and metastasis abilities. It has been reported recently that HKDC1 catalyzes glucose phosphorylation and cellular energy metabolism involving cancer growth and metastasis [23]. In our study, we observed that HKDC1 increased glucose consumption and lactate production, indicating HKDC1 could regulate aerobic glycolysis in LUAD. Chen et al. found that HKDC1 was located on the mitochondrial membrane and regulated the permeability transition pore opening, suggesting a reasonable mechanism for the metabolic effect of HKDC1 [23].

Previous studies have demonstrated that HKDC1 is associated with aggressive phenotype and poor prognosis in HCC [22]. Further, HKDC1 modulates the oxidative stress, apoptosis, proliferation, and metastasis in breast cancer [23]. In our study, we also observed that HKDC1 promoted the proliferation, invasion and EMT capacity in vitro and tumor growth in vivo. These findings demonstrated that HKDC1 could function as an oncogene in LUAD, which provides more evidence that it may serve as a therapeutic target for LUAD.

HKDC1 affects behavioral functions in cancers by different mechanisms. For instance, Chen et al. discovered that HKDC1 knockdown significantly suppressed extranodal nasal-type natural killer/T-cell lymphoma growth through ROS generation and DNA damage [21]. Zhang et al. found that silencing HKDC1 may inhibit cellular proliferation and migration by suppressing Wnt/β-catenin signaling pathway in HCC [22]. In the present study, we determined the oncogenic functions of HKDC1 in LUAD. However, the downstream mechanisms by which HKDC1 influenced LUAD progression were still vague. Thus, we next explored its potential regulatory pathway in LUAD. AMPK is a sensor of energy status that maintains cellular energy homeostasis, which contributes to the regulation of mitochondrial biogenesis and disposal, cell polarity, cell growth and proliferation [31]. mTOR, a central integrator of nutrient and growth factors, is negatively regulated by AMPK, which in turn promotes processes including the cell cycle, cell growth and angiogenesis [32]. AMPK/mTOR signaling pathway is dysregulated in most human cancers and has been considered a promising therapeutic target against cancers [33, 34]. Based on the observation that HKDC1 could promote the aerobic glycolysis in LUAD, we speculated that AMPK/mTOR pathway may mediate the oncogenic functions of HKDC1. To test the effect of HKDC1 on this pathway, western blotting was used to examine the expression of the key factors of the signaling pathway. As expected, HKDC1 overexpression resulted in decreased p-AMPK expression, and increased p-mTOR and p-70S6 expression. More importantly, activating AMPK by AICAR or blocking mTORC1 by rapamycin attenuated the biological effect of HKDC1 on LUAD cells, indicating biological role of HKDC1 was mediated by AMPK/ mTOR signal pathway.

In conclusion, our study demonstrates that HKDC1 plays an oncogenic role in LUAD. Targeting this gene may provide a promising therapeutic target to delay LUAD progression.

## Conclusions

HKDC1 is highly expressed in lung adenocarcinoma (LUAD) and could serve as a prognostic predictor for LUAD patients. Overexpression of HKDC1 promotes the proliferation, migration, invasion, glycolysis, EMT and tumorgenicity of LUAD via activating AMPK/mTOR signaling pathway. Thus, HKDC1 may be a promising therapeutic target of LUAD.

## Data Availability

All data generated or analyzed during this study are included in this published article and its additional files.
